# Mapping compassion in the general adult population: religious and secular compassionate acts in social relationships and organizational culture

**DOI:** 10.3389/fpubh.2025.1704798

**Published:** 2026-01-21

**Authors:** Carlo Lazzari, Paul Crawford, Yasuhiro Kotera

**Affiliations:** 1 International Centre for Healthcare and Medical Education, London, United Kingdom; 2 Institute of Mental Health, University of Nottingham, Nottingham, United Kingdom; 3 Center for Infectious Disease Education and Research, The University of Osaka, Suita, Japan; 4 Department of Social Sciences, Azerbaijan University, Baku, Azerbaijan

**Keywords:** compassion, compassion from others, organizational culture, religion, secularism, society & culture, well-being

## Abstract

**Background:**

Compassion, defined as recognizing suffering and acting to alleviate it, is increasingly acknowledged as a public health asset that enhances resilience, trust, and cooperation. Shown through interpersonal care, ethical leadership, and institutional backing, compassion influences social relationships and organizational culture, although interpretations differ across religious, secular, and cultural contexts. Clarifying how compassion is understood is crucial for fostering inclusive, supportive communities and workplaces, with existing literature linking it to mental health and community-rooted resilience.

**Objectives:**

This study examined how religious and secular ethics shape individuals’ perceptions of compassion received from others, and how these experiences inform social relationships and collective activities. It further explored how compassionate behaviors in workplace settings contribute to organizational climate, an area of growing relevance in public health. Rather than measuring health outcomes directly, the study focused on identifying mechanisms and ingredients for cultivating compassion as a key enabler of public health, with its broader link to well-being supported by existing literature.

**Methods:**

Three rounds of online surveys were conducted with 877 working-age adults in the United States between January and April 2025. Participants represented diverse religious backgrounds, including Christianity, Islam, Buddhism, Hinduism, Judaism, and others. To preserve the conceptual breadth of “organization,” no specific profession was defined, ensuring generalizability across public health contexts. Quantitative analyses employed frequency distributions, chi-square tests, and ANOVA, supplemented by path analysis to aggregate results. Qualitative data were examined through thematic narrative analysis and integrated with set theory models to theorize compassion’s broader role.

**Results:**

Participants reported that compassion was rarely visible in public discourse, particularly in media (χ^2^ = 75.30, *p* < 0.001), despite its perceived importance in healthcare (χ^2^ = 27.11, *p* < 0.001), education, and family life. Receiving compassion in workplaces was strongly linked to improved team cohesion, ethical leadership, and support during personal crises (χ^2^ = 364.32; χ^2^ = 138.29, both *p* < 0.001). Qualitative narratives revealed that compassion was interpreted as empathic acknowledgment, shaped by both religious traditions and secular experiences, and embedded in the social fabric of public and professional life.

**Conclusion:**

This study identifies receiving compassion as a valuable social and organizational resource, positioning it as a potential public health asset. Compassion strengthens social bonds within communities and workplaces, contributing to trust, cooperation, and resilience. Religious frameworks often interpreted compassion as sacred obligation, while secular frameworks emphasized fairness and civic responsibility, together illuminating compassion’s versatility as both spiritual and civic resource. Limitations include reliance on self-reported data, broad framing of “organization,” and a U.S.-based sample. Future research should employ longitudinal and observational designs to validate findings and explore how cultural contexts and intersecting identities shape interpretations of compassion.

## Introduction

### Background/rationale

Compassion, etymologically, is a term originating from the 14th-century “compassioun” meaning “to suffer with another”, or from the Latin “compassionem”, which is formed by the words “com + pati” (pati = “to experience a sense of pity”) and “com” (“together”) (1). Compassion, the ability to recognize suffering and take steps to alleviate it, goes beyond empathy by inspiring supportive actions (2). In public health, it is increasingly regarded as a foundation for resilience, trust, and cooperation, reducing stress and isolation while enhancing mental well-being (3, 4). Scholars define compassion as recognizing suffering, understanding its universality, tolerating discomfort, and being motivated to help. The definition of compassion is also shaped by cultural, ethical, and spiritual traditions, and it is seen as both a moral duty and a practical form of wisdom (5). Most studies focus on how compassion is cultivated—through self-compassion, training, or institutional practices (6), yet little is known about how individuals interpret compassion when received from others. This gap is critical in pluralistic societies, where diverse religious and secular frameworks shape experiences of compassion (7). While existing evidence highlights these factors, little research examines how compassion is perceived when received. This study addresses that gap by positioning compassion within community-rooted resilience and place-based support, which are vital for mental health (8). This study addresses a crucial gap by focusing on how compassion is received and interpreted, rather than just how it is enacted. By placing compassion within community-rooted resilience and public health, it highlights its practical significance for enhancing mental well-being and organizational culture (9). Asano conceptualizes compassion as a multidimensional, culturally embedded process involving three core elements: the recognition of suffering, emotional engagement, and a motivation to alleviate distress (10). His work highlights that compassion is not merely a personal trait but a relational dynamic shaped by cultural norms and interpersonal contexts (10). Through his research on fears of compassion, Asano emphasizes how individuals may struggle with receiving or expressing compassion due to vulnerability, shame, or social expectations, especially in cultures that value emotional restraint (10). As a relational and scientifically relevant concept, compassion promotes well-being, ethical leadership, and collaborative public health efforts (5). Furthermore, compassion intersects with science and spiritual care through shared principles of connectedness, healing, and ethical responsiveness (5). Religious traditions regard compassion as a moral obligation, while psychology considers it a biologically rooted caregiving behavior (5). In this context, compassion from others refers to the experience of being supported by individuals who are both emotionally attuned to one’s distress and motivated to alleviate it through meaningful action (5). For the person receiving compassion, this involves being noticed, understood, and accepted without judgment, while others remain present and responsive to their suffering. Accepting compassion from others is characterized by a sense of emotional safety, where the distress is met with care, tolerance, and practical support, reinforcing the feeling of being valued and connected (5).

A vital distinction exists between compassion from others and the act of receiving compassion. Compassion from others refers to the visible expression of care—acts of kindness, protective support, or empathic presence offered by someone else (11, 12). It is relational and externally expressed, influenced by cultural norms, perceived sincerity, and interpersonal trust (11, 12). This type of compassion can be provided by loved ones, professionals, or strangers, and is usually measured through behavioral indicators or reports from third parties (11, 12). In contrast, receiving compassion pertains to the internal, subjective experience of being cared for; it entails perceiving and emotionally processing another’s compassionate actions, marked by feelings of warmth, safety, and connection during moments of distress (13). This process activates neural systems linked to social bonding and threat reduction and supports psychological resilience, emotion regulation, and attachment wellbeing (14–16).

However, despite extensive research on compassion, there remains limited information on whether people choose secular ethics, common sense, or religion to interpret compassion or to guide their own actions accordingly. To study compassion in detail, we adopted several frameworks. The first theoretical framework is a biopsychosocial-spiritual model in which personal well-being encompasses one’s emotional, physical, mental, spiritual, and social health (17). The second conceptual framework relates to compassion within organizations. Patients, healthcare professionals, and organizations may all gain from compassionate leadership and teamwork, as proposed by the Schwartz Centre for Compassionate Healthcare Framework (18). For example, a compassionate healthcare system can be achieved through seven pledges: compassionate leadership, compassionate education, compassionate valuation and rewards, carer support, patient and family partnerships, compassionate integration into healthcare delivery, and compassionate research and understanding (12). There is a two-way link between compassion and involvement: some clinicians and staff prefer a compassionate work environment because it provides them with a sense of fulfilment (12). Furthermore, compassion is fundamental in helping professions, as spirituality, compassion, religion, and forgiveness impact both the personal well-being of healthcare workers and their empathetic approach to patients (19). The third conceptual framework connects compassion and religion. Compassion is a core, multicultural value that is universally endorsed by all the world’s major faiths. It has been suggested that religion might positively influence health by fostering people’s compassionate attitudes and behaviors towards others (20).

In a study conducted in Japan, compassion from others was measured using a standardised scale. This scale included the following concepts endorsed by a person or respondent: (1) others generating helpful strategies for someone to cope with distress; (2) others focusing their attention on what is likely to benefit a person; (3) others taking actions and doing things that will be advantageous for someone; (4) others treating a person with feelings of support, helpfulness, and encouragement; (5) others reflecting on and understanding a person’s feelings of distress; (6) other people actively motivated to engage and work with a person’s distress when it arises; (7) others noticing and being sensitive to a person’s feelings of distress when they occur; (8) others being accepting, non-critical, and non-judgemental of a person’s feelings of distress; and (9) others being emotionally affected by a person’s feelings of distress (21). Preliminary studies of ours suggest that the cement for collateral sustenance in interprofessional (healthcare) teams is reciprocal empathy and compassion (22). A systematic review of 3,387 trials shows that compassion, through self-compassion, enhances job satisfaction, optimism, and self-care while decreasing burnout and compassion barriers (17). This study also emphasised that self-compassion closely aligns with mindfulness and Buddhist practices, reinforcing its importance in emotional regulation (23). Compassion is also essential in the healthcare profession. For instance, a study examined COVID-19’s mental health impact on Japanese medical workers, emphasizing loneliness, hope, and self-compassion as key factors (24). Another study involving 129 Irish social work students explored compassion and self-compassion in relation to resilience, engagement, and motivation (25). Findings revealed that self-compassion is influenced by personal factors such as age and gender, with resilience and intrinsic motivation serving as key predictors (12). This research highlights the importance of fostering compassion within professional education, suggesting that educators can enhance students’ resilience and motivation to support their well-being (12). Another study found that among United Kingdom education students, compassion, and specifically self-compassion, emerged as the most significant predictor of mental health problems, highlighting its potential role in improving emotional well-being (26). Compassion is widely recognized as a multidimensional construct encompassing emotional, cognitive, and behavioral components that underpin human connection and caregiving roles (27). In the helping professions, such as healthcare, education, and social work, it is considered both a moral imperative and a professional competency (28). While numerous studies have explored how compassion can be taught and cultivated in clinical and educational settings (29, 30), there remains a gap in understanding how individuals receive and interpret compassion from others, particularly within organizational and societal contexts (31, 32).

This interpretive gap is especially relevant when considering the frameworks people use to make sense of compassionate acts. Religious traditions often provide structured moral narratives that frame compassion as a spiritual duty (33), while secular reasoning, rooted in empathy, reciprocity, or human rights, offers alternative, intuitive pathways for compassionate behavior (34). However, empirical research on how individuals draw upon either religious or secular frameworks to guide their interpersonal compassion remains limited, particularly in pluralistic and multicultural societies.

The decision to start this wave of research with a sample of 877 working-age adults from the United States was based on both methodological and contextual reasons. The United States presents a uniquely pluralistic religious and secular environment, making it an ideal location for examining received compassion across different belief systems. Its healthcare system widely incorporates Clinical Pastoral Education (CPE) and spiritual health practitioners, whose roles are specifically focused on compassionate care for patients, families, and staff (35, 36). This institutional embedding of compassion supports our study’s aim to examine compassion as a public health resource. While foundational literature from the United Kingdom and Japan was used to establish theoretical grounding—particularly Gilbert’s evolutionary model and Asano’s cross-cultural insights—these frameworks were chosen for their conceptual relevance rather than geographic alignment. Subsequent waves will expand to other regions, enabling comparative analysis and cultural adaptation of the model. We utilized the STROBE (Strengthening the Reporting of Observational Studies in Epidemiology) checklist to structure the manuscript sections (see Appendix) (37).

### Objectives and hypotheses

**Figure 1:**
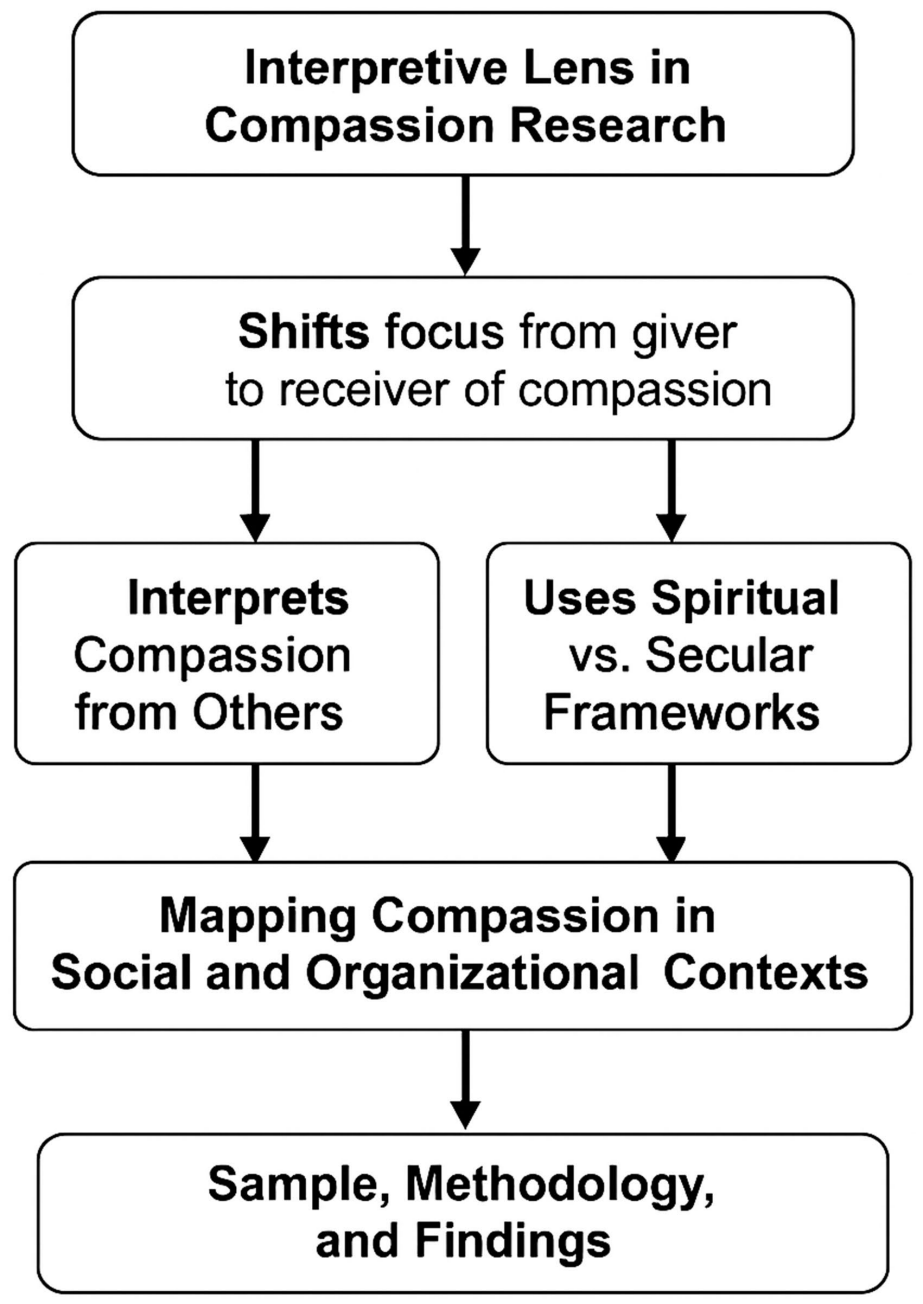
This study shifts focus from giving to receiving compassion, exploring how individuals interpret compassionate acts through religious, secular, or intuitive frameworks. It maps compassion in social and organizational contexts, revealing implications for public health, ethics, and institutional culture.

The study aimed to fill a key gap in compassion research by exploring how compassion is understood when received, rather than how it is developed or expressed. Specifically, the objectives follow (see Figure 1).

1. Interpretive frameworks: Assess whether individuals rely on religious teachings, secular ethics, or intuitive reasoning when perceiving compassion.
2. Contextual reception: Examines how compassionate behavior is recognised and understood across family, workplace, healthcare, and community settings.
3. Pluralistic relevance: Examines how various belief systems in modern societies influence the experience and significance of compassion.
4. Mapping compassion: Recognizes and interprets compassionate acts within social, organizational, and community settings as relational mechanisms that promote resilience and place-based support essential for personal well-being.
5. Public health and institutional implications: Examine how compassion, as perceived and understood, indirectly influences well-being, organizational culture, and the embedding of compassionate practices.

Hypotheses:

- H1: Individuals interpret compassion through different frameworks (religious, secular, or intuitive), with measurable differences across demographic groups.
- H2: Recognition and understanding of compassionate behavior vary notably across family, workplace, healthcare, religious, secular, and community settings.
- H3: Diverse belief systems in contemporary societies influence how compassion is perceived in terms of meaning and relevance.
- H4: Compassionate acts serve as relational mechanisms that strengthen resilience and place-based support, especially within organizational and community contexts.
- H5: Interpreting compassion indirectly enhances individual well-being, boosts community resilience, and promotes the embedding of compassionate practices within public health and organizational culture.

## Methodology

This study employed a mixed-methods explanatory design, combining quantitative surveys with qualitative responses to explore how religious and secular ethics influence perceptions of compassion. Quantitative data revealed broad trends, while qualitative insights provided clarification and depth, following Creswell’s explanatory sequential model. Based on pragmatism, the approach focused on real-world problem-solving by merging statistical analysis with thematic exploration (38). Ontologically, compassion was regarded as both a personal experience and a socially constructed phenomenon influenced by cultural and ethical contexts. Epistemologically, the study employed a pluralistic approach, utilizing various methods to identify both general patterns and individual meanings. Axiologically, the research was guided by principles of relational ethics and inclusivity, prioritizing transparency, reciprocity, and fairness (39). Compassion itself functioned as both the focus of the inquiry and a methodological guideline, ensuring the study was conducted in a respectful and culturally sensitive manner (40–43).

### Research question

How do individuals interpret compassion received from others, whether through religious or secular frameworks—, and in what ways can these interpretations shape the development of individual and community-based resilience, organizational culture, and place-specific practices that frame compassion as a public health resource?

## Methods

### Study design and setting

This is a mixed-method explanatory research study conducted between January and April 2025. We used three anonymous online surveys in three separate phases, gathering data from an electronic and public platform: SurveyMonkey. Data included both numerical and qualitative information. Quantitative analysis involved Chi-square χ2 (Goodness of Fit Index = GFI), t-test statistics, one-way ANOVA F, and cross-correlations. Since the direction of the effect was unknown, we employed two-tailed *p*-values. Data were collected using close-ended questions on Likert scales. Qualitative analysis was conducted through thematic analysis, identifying themes and subthemes from open-ended responses. Medcalc and NVIVO facilitated the quantitative and qualitative analyses, respectively. Quantitative results were deemed statistically significant when rejecting the null hypothesis at an alpha level of *p* < 0.05. Effect sizes were measured using Cohen’s *w* or *d* coefficients. Data were initially gathered and elaborated for frequency distribution via an online survey engine. The survey distribution was anonymous and outsourced to a third-party specialized in collecting customer surveys, to address ethical and practical issues such as biases, locality, religious beliefs, geographical location, and personal matters related to faith, spirituality, and experiences in the subject. We employed three different surveys as assessment tools, each refined across three successive waves. This iterative process allowed us to enhance clarity, validity, and contextual relevance at each stage, ensuring that the final instruments captured nuanced interpretations of compassion across religious, secular, interpersonal, and organizational settings. By progressively refining the surveys, we improved both methodological robustness and the reliability of the findings, aligning the tools with the study’s goal of exploring how compassion is perceived and understood, rather than merely enacted.

### Participants

The surveys used in this study were designed and conducted by the authors as part of original data collection, rather than relying on secondary datasets or pre-existing SurveyMonkey databases. Administered online to a United States sample of 877 working-age adults through random and opportunistic recruitment, the survey instrument focused on how individuals perceive and interpret compassion received from others, examining religious and secular frameworks, interpersonal relationships, and organizational contexts. The primary aim was to investigate the mechanisms and interpretive lenses through which compassion is understood, not to measure direct health or well-being outcomes. To address the potential disconnect with public health, the study has been reframed to emphasize compassion as a social and organizational resource and a potential public health asset, with its link to health and well-being supported by existing literature rather than direct measurement. We selected working-age Americans because the United States offers a pluralistic context where diverse religious, secular, and cultural frameworks shape how compassion is interpreted. This group is embedded in families, workplaces, and communities, making their perspectives highly relevant to public health, organizational practice, and resilience. Their diversity and accessibility through online recruitment provided a robust sample to explore how compassion is received and understood in everyday social and professional settings. The study took place from January to April 2025.

#### Sampling

We used an opportunistic and anonymous sample of the general population in the USA, voluntarily participating in an anonymous online survey on key aspects of religion, compassion, well-being, and social relationships.

### Variables

In organizing the study, the following elements were identified to ensure clarity and logical sequence.

1. Outcomes (dependent variables): the primary outcomes included perceptions of compassion across individual, organizational, and religious domains, alongside well-being indicators such as psychological distress, resilience, belonging, and social cohesion. Secondary outcomes encompassed organizational performance measures, including team cohesion, morale, job satisfaction, and prosocial behaviors. These were assessed through survey responses, validated compassion scales, and thematic coding of qualitative data.
2. Exposures (independent variables): key exposures included workplace and societal dynamics such as supportive relationships and collaborative practices, religious principles like the Golden Rule, charity, patience, and forgiveness, media exposure and educational promotion of compassion, and healthcare organizational structures, particularly staffing levels and administrative burdens.
3. Predictors: demographic factors such as age, gender, religious affiliation, and education were considered, alongside ethical orientation and relational context (team-based practices and moral reasoning). The public health setting of practice (SoP), including community, workplace, and healthcare institutions, was also regarded as a predictor.
4. Potential confounders: socioeconomic status, cultural background, prior mental health conditions, organizational size and resources, and media exposure unrelated to compassion were identified as potential confounders and accounted for in analysis.
5. Effect modifiers (interaction variables): age was examined as a moderator of the relationship between compassion and workplace cohesion, religious affiliation as a modifier of compassion’s impact on ethical leadership, and organizational context as a factor influencing the effect of compassion on well-being outcomes.

### Bias

Bias was addressed by identifying and mitigating potential sources at each stage of the study. Selection bias was minimized through clear inclusion and exclusion criteria and transparent recruitment procedures. Measurement bias was reduced by triangulating survey data with qualitative coding. Confounding bias was managed by adjusting for demographic and contextual variables such as age, religious affiliation, socioeconomic status, and organizational size. Analytical bias was limited through sensitivity analyses and the use of interaction terms to test effect modification.

### Descriptive quantitative analysis

In the quantitative strand, data were analyzed using the chi-square goodness-of-fit test (GFI) to identify differences in response percentages and assess their statistical significance. An alpha threshold of *p* = 0.05 was applied to determine meaningful variation across categories. Additionally, cross-correlational analysis was conducted to explore associations between variables, providing further insight into how different factors influenced interpretations of compassion. Quantitative data comprised answers to closed-ended questions.

#### Path analysis

Path analysis explored how multiple variables could be combined to form a cohesive framework for understanding perceived compassion in society and organizations, as well as its attribution to religious or secular frameworks. Participants (adults aged 18 and above) responded to structured items and open-ended prompts concerning compassion in workplace or social contexts. Thematic coding was applied to qualitative responses, while standardised path coefficients were calculated using structural equation modelling (SEM) for quantitative variables to evaluate directional relationships between variables such as age, gender, religious orientation, and compassion-related outcomes. Path analysis adhered to guidelines from Kline, ensuring model fit and clarity (44). Path analyses were conducted using AMOS v.26 (45). RMSEA (Root Mean Square Error of Approximation) is a commonly used index to evaluate the goodness-of-fit in structural equation modelling, including path analysis. It measures how well the proposed model fits the population covariance matrix, accounting for model complexity (46). For our path analysis, RMSEA was utilized to assess the structural validity of the model connecting demographic factors, media perception, educational attitudes, and experiential frequency to moral reasoning. A value below 0.06 indicated a good fit, suggesting that the model effectively captured the observed relationships and supported the robustness of compassion as a teachable and psychologically significant construct.

### Statistical power and effect size justification

To evaluate associations between categorical and continuous variables, the study employed statistical procedures guided by the Power*G framework (47, 48). All analyses were configured to achieve 80% power (1 − *β* = 0.80) at a conventional alpha level of 0.05, ensuring robust detection of meaningful effects while minimizing the risk of Type I error. The chi-square goodness-of-fit (GFI) test was used to examine whether the observed distributions of Likert-type responses deviated from theoretical expectations. A medium effect size (w = 0.5), aligned with Cohen’s (49) recommendations, guided the selection of critical chi-square values, which were χ^2^ = 5.9 for 2-point and 3-point scales, and χ^2^ = 7.8 for 4-point scales. The analysis incorporated data from three surveys (*N* = 318, 244, and 285, respectively), each exceeding the minimum required sample size to reliably detect medium effects. In accordance with Psychometrica conventions, effect sizes for chi-square analyses were interpreted using Cohen’s w, with small effects ranging from 0.1 to 0.4, intermediate from 0.5 to 0.7, and large effects defined as >0.8 (50). To investigate differences in group means across continuous outcomes, one-way ANOVA was employed. A medium effect size (*f* = 0.39) was specified following Cohen’s guidelines. Sample size requirements were determined based on the number of predictors: *n* = 66 for two predictors, *n* = 75 for three predictors, and *n* = 83 for four predictors, while maintaining the desired statistical power threshold. Effect sizes from ANOVA tests were calculated and interpreted using Cohen’s *d*, as recommended by Psychometrica (51). Small effects ranged from 0.1 to 0.4, medium effects from 0.5 to 0.7, and large effects exceeded 0.8. By applying these calibrated thresholds and analytic conventions, the statistical design was sensitive to practical implications and consistent with best practices in psychological and social research.

### Descriptive qualitative analysis

#### Thematic analysis

In the qualitative strand of this study, we analyzed participants’ open-ended survey responses using Braun and Clarke’s six-phase framework for thematic analysis. This process began with familiarization, where responses were read and re-read to capture initial impressions, followed by systematic coding in which meaningful segments of text were labeled to reflect their content. Codes were then organized into broader themes, such as compassion as understanding, supportive relationships, and collective response, with subthemes identified to capture finer distinctions. Each theme was reviewed against the dataset to ensure coherence and then clearly defined to represent its boundaries. This iterative approach balanced flexibility with rigor, enabling us to identify patterned meanings while remaining attentive to contextual nuance. The resulting themes provided insight into how compassion was interpreted across diverse settings, including religious, secular, interpersonal, and organizational domains (52).

#### Set theory analysis

To complement this thematic approach, we applied set theory as a formal lens to represent relationships among themes (53). Each thematic domain was treated as a set—for example, C₁ (understanding), C₂ (supportive relationships), and C₃ (collective response). The union (C₁ ∪ C₂ ∪ C₃) represented the full conceptual space of compassion, while the intersection (C₁ ∩ C₂ ∩ C₃) highlighted shared attributes such as empathy, trust, and care. This framework allowed us to model overlaps and distinctions across contexts with clarity and reproducibility. Religious (R) and secular (S) orientations were considered conditioning sets that influenced how compassion was expressed, shaping the thematic landscape without altering its core traits. Set Theory Analysis extended this representation by modeling directional relationships between latent constructs, expressed as functions (*f*: Lᵢ → Lⱼ), to illustrate how one dimension of compassion influenced another. The symbol L denotes a latent construct, or latent variable. Latent constructs are abstract dimensions that cannot be directly observed but are inferred from patterns in the data. In this study, such constructs included compassion as understanding, supportive relationships, and collective response—each distilled from the thematic analysis of participants’ responses. The subscripts i and j simply distinguish between different constructs, with (L_i_) representing the source construct (for example, compassion as understanding) and (L_j_) representing the target construct (such as supportive relationships). The function (*f*) expresses a path or directional relationship between constructs, showing how variation in one latent dimension influences another. In plain terms, L refers to an abstract theme identified in the analysis, and the mapping (*f*: Lᵢ → Lⱼ) models how one construct affects or predicts another. These operations formally represent how constructs relate across contexts and enable reproducible conceptual mapping (51, 54). Set theory modelling provides a structured framework for qualitative research, moving beyond description to systematic explanation of complex phenomena. By treating attributes and outcomes as sets, researchers can analyse how conditions combine and interact, especially in contexts of conjunctural causality. Its main application is Qualitative Comparative Analysis (QCA), which uses Boolean logic to identify necessary and sufficient conditions, explore equifinality, and build rigorous models. This enables outcomes such as policy success or organizational change to be understood as configurations of factors rather than isolated variables (51, 55–57).

### Statistical software

We used Medcalc (83) for general statistical analysis. G*Power (47, 48) calculator for sample size and statistical power. Psychometrica (50) was used for the calculation of the effect size. Chi-square GFI was obtained using the Statology program (58). The Chi-Square power calculator was accessed via the Statistic Kingdom (59). Qualitative analysis was conducted with NVIVO (60). Path analysis was conducted with AMOS (61). Set theory and writing was helped by Wolfram Alpha (62).

### Triangulation methods

We employed a convergent mixed methods triangulation design to integrate quantitative and qualitative strands. Quantitative analyses (chi-square, correlations, path analysis) and qualitative thematic analysis (Braun and Clarke) were conducted in parallel. Results were then mapped comparatively, aligning statistical patterns with thematic domains to identify convergence, divergence, and complementarity. Using set theory integration, thematic overlaps and distinctions were systematically cross-referenced with quantitative associations. Religious and secular conditioning frameworks were examined across both strands to assess belief-system influences on compassion. Finally, meta-inference synthesized findings into a unified interpretation, where statistical significance reinforced thematic salience and qualitative nuance explained quantitative variation (38, 63).

### Ethical considerations

This study did not require formal ethical approval due to its design and data-handling procedures. The data collection was conducted by an independent international research company using opportunistic sampling, with responses fully anonymous and no personally identifiable information collected. In line with guidance from the UK Health Research Authority, the University of Aberdeen’s SERB, and the University of Nottingham’s ethics flowchart, anonymous surveys that do not involve sensitive data or vulnerable populations are usually exempt from formal review. The survey focused on attitudinal and perceptual variables related to compassion, without probing health, trauma, or protected characteristics. Data collection complied with GDPR and institutional privacy protocols, and the research adhered to the Declaration of Helsinki (2013), ensuring respect for autonomy, confidentiality, and beneficence. Participants were informed of the study’s purpose, voluntary participation, and the anonymity of responses, with no deception, coercion, or follow-up contact. This approach aligns with best practices for low-risk behavioral research (64–67).

### Surveys methods

PICOS (Population, Intervention, Comparison, Outcome, and Settings/Study Design) frameworks were used to organize the survey data.

#### Survey no. 1 – compassion, common sense, and religion

This survey investigated how adults aged 18 and above (population) perceive compassion when it is represented in media, education, and broader social systems (intervention). Responses were compared across religious and secular frameworks (comparison) to understand how belief systems influence attitudes toward compassion. The outcomes (outcome) examined whether compassion is viewed as rooted in religion, secular ethics, sufficiently represented in media, necessary in areas such as healthcare, education, and politics, and whether it should be explicitly taught. Conducted within societal contexts including media, education, and public discourse (Settings), the study indicates that compassion is a socially constructed and ethically guided principle. The two aspects of survey no. 1—working population and religious affiliations—demonstrate how compassion is interpreted both in daily life and across cultural traditions. Although not explicitly framed as public health research, the findings imply that compassion plays a role in public and mental health, resilience, and social cohesion, offering valuable insights into the ethical foundations that underpin well-being and healthier communities.

#### Survey no. 2 – compassion in organizations

This survey examined how adults aged 18 and over working in team-based environments (population) perceive compassion in organizational relationships and workplace culture (intervention). It compared responses across demographics, professional backgrounds, and crisis scenarios (comparison) to identify patterns in valuing and practising compassion. Outcomes (outcome) highlighted its influence on team satisfaction, engagement, and performance, demonstrating how relational empathy enhances workplace effectiveness. Conducted within organizational contexts such as teams and companies (settings), the study suggests that compassion is a vital cultural element. Beyond workplace success, findings indicate compassion supports mental well-being, resilience, and social cohesion, linking organizational ethics with public health.

#### Survey no. 3: perceived compassion in organizations and role of religion or common sense

**Figure 2:**
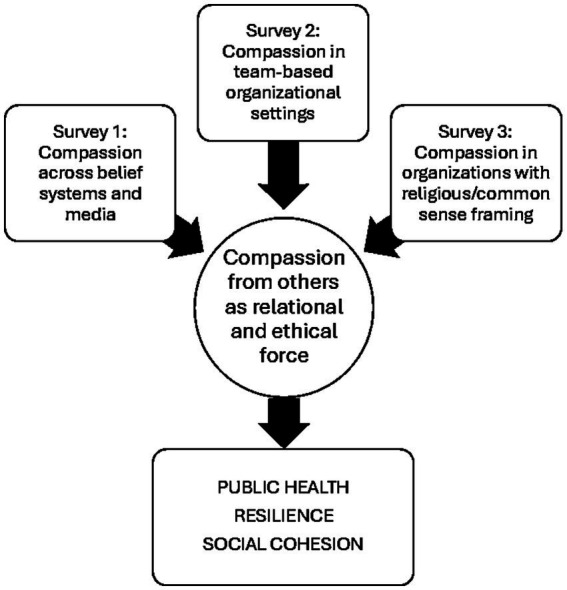
Survey no. 1 examined compassion across belief systems and social contexts; survey no. 2 explored compassion in organizational culture; survey no. 3 investigated compassion in workplace settings shaped by religion or common sense. Together, they converge on the role of belief and context in valuing compassion, leading to shared perceptions in social and organizational environments and ultimately linking compassion to public health outcomes such as resilience, well-being, and social cohesion.

This study explored how adults aged 18 years and older working in various types of organizations (population) perceive and interpret compassion within organizational settings (intervention). It examined differences in responses based on demographic characteristics, religious orientation, and workplace experiences (comparison), enabling a detailed understanding of how personal and contextual factors influence attitudes towards compassion. The study aimed to provide insights into the role of compassion in team dynamics and the impact of religious versus secular guidance on workplace behavior (outcome), positioning compassion as both a relational and ethical concept. Carried out across unspecified workplace organizations of any kind (settings), the research contributes to a broader understanding of how compassion operates within diverse professional environments, informing leadership practices, team cohesion, and organizational culture (Figure 2).

## Results

### Results from survey no. 1: compassion, common sense, and religion

The research hypothesis was that people’s views on compassion vary depending on their background and professional setting, with broad support for making compassion more visible and integrating it into key social areas, such as the media and education. The survey (population: *N* = 318) of the adult population, regardless of religion, examined perceptions of compassion across gender, age, and professional contexts, revealing statistically significant trends. The gender distribution was balanced (male: 45.91%, female: 54.09%, *p* = n.s.), indicating no significant difference. However, age-related differences were notable (χ^2^ = 73.37, *p* < 0.001, w = 1.09), with the largest group aged 30–44 (37.42%), while religious affiliation also showed significant variation (χ^2^ = 223.81; *p* < 0.001; w = 0.6). A majority (50.63%) believed that compassion is underrepresented in modern media (χ^2^ = 75.30, *p* < 0.001, w = 1.11), highlighting concerns about public awareness. Healthcare was seen as the field most needing compassion (73.90%), followed by education (66.98%), family (56.92%), community services (50.00%), and politics (47.48%) (χ^2^ = 27.11, *p* < 0.001, w = 0.34). Notably, 71.38% supported explicit compassion education (χ^2^ = 370.57, *p* < 0.001, w > 1.0), indicating strong consensus. Daily experiences of compassion varied: 44.65% reported occasional exposure, while 34.28% reported frequent exposure (χ^2^ = 108.54, *p* < 0.001, w = 1.4). Views on the origin of compassion were evenly divided: 28.93% linked it to religious teachings, 28.62% to common sense, and 36.79% to both equally (χ^2^ = 131.86, *p* < 0.001, w = 1.6) (Table 1).

**Table 1:** Survey no. 1 – compassion, common sense, and religion (multiple-choice questions).

Survey no. 1 (*N* = 318)	Answer choice	Responses (%)	*N*	χ^2^ (GFI) (significance *p*)	Effect size Cohen’s w
Gender	Male	45.91%	146	n.s.	0.00
	Female	54.09%	172		
Age	18–29	10.06%	32	73.37 (<0.001)	1.09
	30–44	37.42%	119		
	45–60	36.16%	115		
	>60	16.35%	52		
Religious affiliation	Christianity	39.85%	127	131.13 (*p* < 0.001)	0.6
	Islam	12.43%	40		
	Buddhism	16.45%	52		
	Hinduism	10.96%	35		
	Judaism	12.25%	39		
	Other	8.5%	25		
Do you believe that compassion is adequately represented in modern media?	Yes, it is well represented	37.74%	120	75.30 (<0.001)	1.11
	No, it needs more representation	50.63%	161		
	Unsure	11.64%	37		
Select all that apply: In which of the following areas do you think compassion is most needed today?	Healthcare	73.90%	235	27.11 (<0.001)	0.34
	Education	66.98%	213		
	Politics	47.48%	151		
	Community services	50.00%	159		
	Family	56.92%	181		
Do you think compassion should be taught explicitly as part of educational curricula?	Yes	71.38%	227	370.57 (<0.001)	>1.0
	No	12.58%	40		
	Only as part of religious studies	4.09%	13		
	Only as part of ethics or philosophy classes	11.95%	38		
How often do you encounter acts of compassion in your daily life?	Very often	34.28%	109	108.54 (<0.001)	1.4
	Occasionally	44.65%	142		
	Never	1.57%	5		
In your opinion, does compassion derive more from religious teachings or from common sense?	Mostly from religious teachings	28.93%	92	131.86 (<0.001)	1.6
	Mostly from common sense	28.62%	91		
	Both equally	36.79%	117		
	Neither	5.66%	18		

### Path analysis

Our path analysis revealed a clear pathway linking demographic identity to attitudes about compassion, with age and religious affiliation identified as key predictors. Older respondents were more likely to see compassion as underrepresented in media (β = 0.42) and rooted in religious teachings (β = 0.33), while media perception served as a central mediator, predicting support for incorporating compassion into curricula (β = 0.51) and influencing perceptions of its societal importance (β = 0.36). Support for compassion education correlated with increased recognition of compassionate acts in daily life (β = 0.48), which in turn promoted balanced views that integrated religious and common-sense origins (β = 0.55). Religious affiliation significantly impacted multiple domains, including media representation (β = 0.42), perceived societal need (β = 0.38), support for curricula (β = 0.49), daily interactions (β = 0.44), and conceptual origins (β = 0.51), affirming its role in shaping the meaning and value of compassion. Gender was excluded due to non-significance. Survey no. 1 (N = 318) reinforced these findings, showing that age and religious background consistently shape perceptions of compassion. Model fit was strong (RMSEA ≈ 0.05), supporting the structural validity of the proposed compassion pathway.

### Qualitative text analysis in survey no. 1 (open-ended questions)

**Figure 3:**
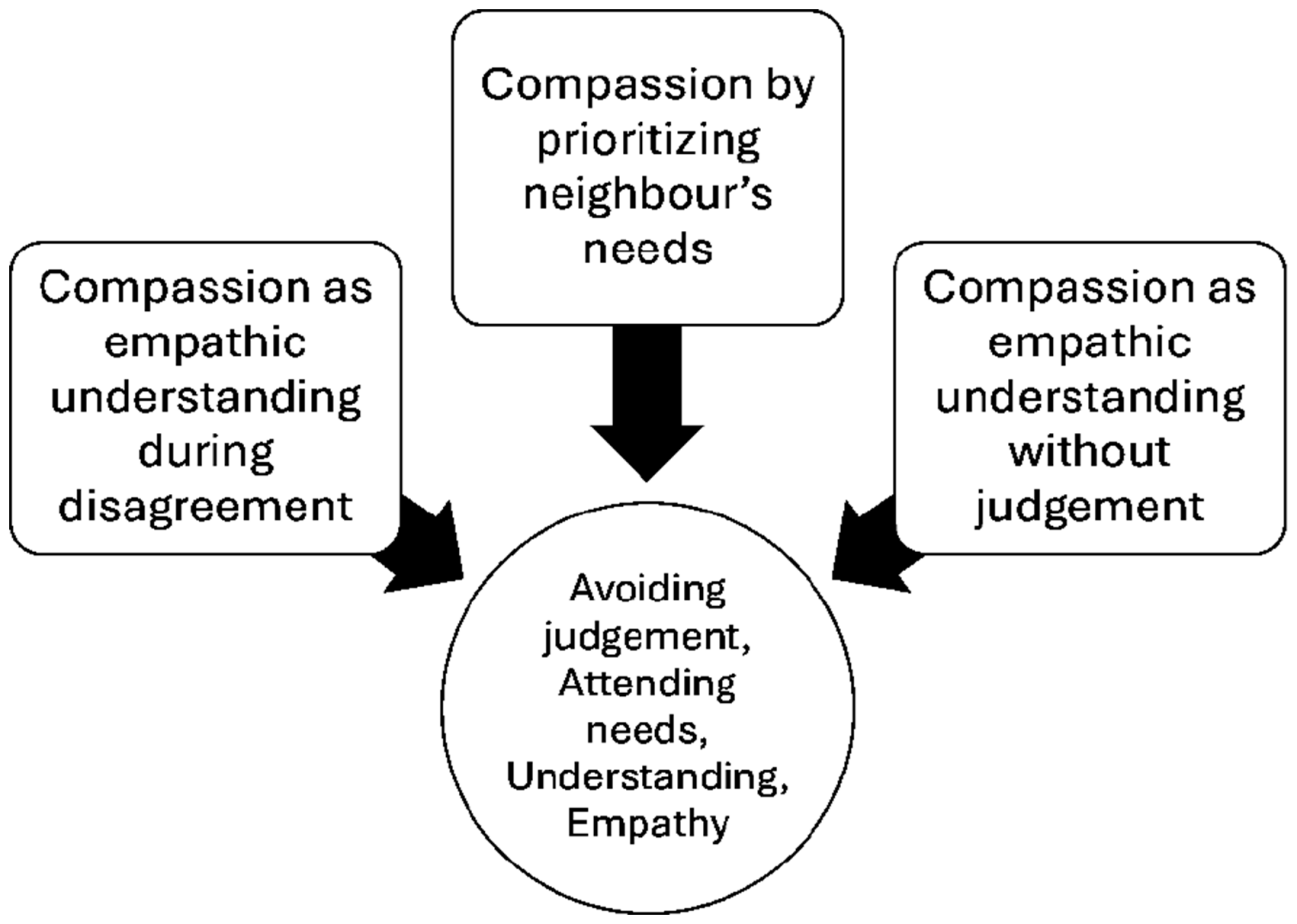
Mapping themes of the qualitative analysis.

In Survey No. 1, participants were asked directly: “How do you define compassion in your own words?” Their responses revealed three distinct but interconnected themes. The first theme, compassion as empathic understanding during disagreement, emphasized the ability to remain empathetic even when perspectives diverge, exemplified by the statement “empathic understanding even if you don’t agree.” The second theme, compassion as empathic understanding without judgment, highlighted openness and acceptance, where understanding is offered without criticism, illustrated by the response “to show understanding to another without judgment.” The third theme, compassion by prioritizing neighbors’ needs, reflected a more action-oriented dimension, portraying compassion as selflessness and care for others, expressed in the phrase “putting the needs of someone else before your own.” Taken together, these themes demonstrate that compassion is understood both as an inner disposition—marked by empathy and non-judgment—and as an outward practice of prioritizing others’ well-being, bridging differences, fostering acceptance, and motivating altruistic action (Figure 3).

### Set theory model from the qualitative survey no. 1

In this study, the Universe of Discourse (U) included all participant definitions of compassion across media, education, belief, and society. Three sets were established: A = empathic understanding during disagreement, B = empathic understanding without judgment, and C = prioritizing others’ needs. Their intersections highlighted key relational dimensions: A ∩ B reflected emotional regulation in disagreement versus judgment; B ∩ C emphasized non-self-centeredness and moral regard; A ∩ C revealed ethical tensions between personal beliefs and altruism. The union A ∪ B ∪ C represented a combined definition of compassion as empathic understanding + non-judgment + altruistic prioritisation. A cyclic path (A → B → C → A) illustrated how empathy in disagreement promotes non-judgment, which enables altruism, which then reinforces empathy. Formally, *f*:{A, B, C} → {B, C, A} with *f*(A) = B, *f*(B) = C, and *f*(C) = A. This set-theoretic model demonstrated that received compassion forms a dynamic relational loop, strengthening social relationships and organizational culture. While health outcomes were not directly measured, existing literature positions compassion as a public health asset, suggesting that A ∪ B ∪ C enhances emotional safety, supports mental health, and fosters inclusive environments.

### Details of survey no. 1 and cross-sectional study (compassion, common sense, and religion)

The survey explored the connection between compassion and religious teachings across major world religions. Among participants, 40.83% believed compassion arises equally from religious teachings and common sense, while 34.86% attributed it mainly to religious teachings. The variation in responses was statistically significant (ANOVA F = 6.43, *p* = 0.012, d = 0.20). Views on how modern media portrays compassion varied; 51.38% felt it required more visibility, with Buddhism (65.56%), Hinduism (68.33%), and Judaism (61.19%) showing strong agreement (ANOVA F = 4.29, *p* = 0.033, d = 0.16). Healthcare was identified as the area where compassion is most needed (74.31%), followed closely by education (66.06%) and family (61.93%). Differences between religious groups were not statistically significant (ANOVA F = 0.68, *p* = 0.41, d = 0.06), indicating consensus. A large majority (76.23%) supported explicitly teaching compassion in educational curricula, especially within Islam (85.29%) and Hinduism (80%). However, only 5.50% felt it should be limited to religious studies, while 10.09% preferred ethics or philosophy courses (ANOVA F = 0.77, *p* = n.s., d = 0.07; Table 2).

**Table 2:** Survey no. 1 – cross-sectional study (*N* = 318) (multiple-choice questions).

		Which world religion do you think emphasizes compassion as a core tenet?							
Question	Answer	Christianity	Islam	Buddhism	Hinduism	Judaism	Other	ANOVA F (*p*)	Effect size *d*
In your opinion, does compassion derive more from religious teaching or common sense?	Mostly from religious teachings	76 (34.86%)	23 (33.82%)	16 (17.78%)	10 (16.67%)	21 (31.34%)	8 (6.82%)	6.43 (0.012)	0.20
	Mostly from common sense	48 (22.02%)	17 (25%)	27 (30%)	21 (35%)	15 (22.39%)	27 (61.36%)		
	Both equally	89 (40.83%)	24 (35.29%)	38 (42.22%)	24 (40%)	24 (35.82%)	10 (22.73%)		
	Neither	5 (2.29%)	4 (5.88%)	9 (10%)	5 (8.33%)	7 (10.45%)	4 (9.09%)		
Do you believe that compassion is adequately represented in modern media?	Yes, it is well represented	83 (30.07%)	35 (51.47%)	19 (21.11%)	13 (26.87%)	18 (26.87%)	5 (11.36%)	4.29 (0.033)	0.16
	No, it needs more representation	112 (51.38%)	27 (39.71%)	59 (65.56%)	41 (68.33%)	41 (61.19%)	32 (72.73%)		
	Unsure	23 (10.55%)	6 (8.82%)	12 (13.33%)	6 (10%)	8 (11.94%)	7 (15.81%)		
In which of the following areas do you think compassion is most needed today?	Healthcare	162 (74.31%)	51 (75%)	74 (8.22%)	49 (81.67%)	57 (85.07%)	36 (81.82%)	0.68 (n.s.)	0.06
	Education	144 (66.06%)	57 (83.82%)	67 (74.44%)	45 (75%)	55 (82.09%)	30 (61.18%)		
	Politics	103 (47.25%)	44 (64.71%)	48 (53.33%)	30 (63.33%)	41 (61.19%)	25 (56.82%)		
	Business	77 (35.32%)	31 (45.59%)	42 (46.67%)	29 (48.33%)	32 (47.67%)	22 (50%)		
	Community services	115 (52.75%)	42 (60.29%)	67 (74.44%)	43 (71.76%)	47 (70.15%)	23 (52.27%)		
	Family	135 (61.93%)	39 (57.35%)	62 (68.89%)	44 (73.33%)	45 (67.16%)	29 (65.91%)		
Do you think compassion should be taught explicitly as part of educational curricula?	Yes	164 (76.23%)	58 (85.29%)	61 (67.78%)	48 (80%)	50 (74.63%)	24 (54.55%)	0.77 (n.s.)	0.07
	No	20 (9.17%)	6 (8.82%)	8 (8.89%)	3 (5%)	6 (8.96%)	10 (22.73%)		
	Only as part of religious studies	12 (5.50%)	2 (2.94%)	5 (5.56%)	3 (5%)	3 (4.48%)	1 (2.27%)		
	Only as part of ethics or philosophy classes	22 (10.09%)	2 (2.94%)	16 (17.78%)	6 (10%)	8 (11.94%)	9 (24.95%)		

### Path analysis

The model revealed a structured pathway linking religious affiliation to the conceptual, perceptual, and pedagogical aspects of compassion. Religious affiliation significantly predicted attributing compassion to religious teachings over common sense (β = 0.20, ANOVA F = 6.43, *p* = 0.012, d = 0.20), and perceptions of its underrepresentation in modern media (β = 0.16, ANOVA F = 4.29, *p* = 0.033, d = 0.16), with Buddhism, Hinduism, and Judaism showing the strongest agreement. Support for including compassion in curricula was modestly predicted (β = 0.07), with Islam (85.29%) and Hinduism (80%) showing the highest endorsement. Conversely, prioritization of compassion across societal domains (healthcare, education, family) was consistent across groups and not significantly predicted (ANOVA F = 0.68, *p* = 0.41, d = 0.06), while preferences for teaching methods showed no variation (ANOVA F = 0.77, n.s., d = 0.07). Overall, religious affiliation influenced conceptual framing and media critique but had a limited impact on domain prioritisation or pedagogical preferences, reinforcing compassion’s shared societal importance. Model fit was strong, with RMSEA ≈ 0.05 (values < 0.06 indicating good fit), supporting the structural validity of the pathway.

### Result from survey no. 2: compassion in organizations

The research hypothesis was that compassion is viewed as a crucial factor in effective organizational relationships, especially in team dynamics and crisis management among mid-career professionals across various sectors. The survey (population: *N* = 274) investigated perceptions of compassion within organizational relationships, uncovering statistically significant trends. Gender distribution was balanced (male: 48.91%, female: 51.09%, *p* = n.s.), with the largest age group being 30–44 (41.61%), closely followed by 45–60 (40.51%), indicating an even distribution among mid-career professionals (χ^2^ = 113.18, *p* < 0.001, w = 1.67). Religious affiliations were varied, with Christianity accounting for the largest share (40.88%), then Buddhism (15.33%), Islam (13.14%), Hinduism (10.22%), Judaism (11.68%), and Other (8.76%) (χ^2^ = 119.90, *p* < 0.001, w = 0.66). Compassion was regarded as highly important in team relationships, with 59.85% rating it as “very important” and 32.12% as “important” (χ^2^ = 364.32, *p* < 0.001, w > 3.0). Regarding organizational compassion during crises, 41.24% felt their organization “always” displayed compassion, while 23.72% reported “often” and 26.28% “sometimes” (χ^2^ = 138.29, *p* < 0.001, w = 2.01). Compassion was widely recognised as a key skill in team building, with 53.65% strongly agreeing and 35.40% agreeing (χ^2^ = 306.40, *p* < 0.001, w > 3.0). Essential components of compassionate teamwork included active listening (80.66%), empathy (65.69%), and supportive communication (62.41%) (χ^2^ = 85.31, *p* < 0.001, w = 1.34). Team compassion satisfaction was high, with 42.34% “very satisfied” and 35.77% “satisfied” (χ^2^ = 190.48, *p* < 0.001, w = 3.021). Notably, 59.49% strongly agreed that compassion enhances team performance (χ^2^ = 363.44, *p* < 0.001, w > 3.0), underlining its vital role in workplace effectiveness (Table 3).

**Table 3:** Survey no. 2 – compassion in organizations (multiple-choice questions).

Survey no. 3 (*N* = 274)	Answer choice	Responses (%)	N	χ^2^ (GFI) (significance *p*)	Effect size Cohen’s *w*
Gender:	Male	48.91%	134	n.s.	
	Female	51.09%	140		
Age	18–29	8.39%	23	113.18 (<0.001)	1.67
	30–44	41.61%	114		
	45–60	40.51%	111		
	>60	9.49%	26		
Religious affiliation	Christianity	40.88%	112	119.90 (*p* < 0.001)	0.66
	Islam	13.14%	36		
	Buddhism	15.33%	42		
	Hinduism	10.22%	28		
	Judaism	11.68%	32		
	Other	8.76%	24		
How important do you believe compassion is in team relationships?	Very important	59.85%	164	364.32 (<0.001)	>3.0
	Important	32.12%	88		
	Moderately Important	6.57%	18		
	Slightly Important	1.46%	4		
	Not important	0.00%	0		
In moments of crisis or complex situations, do you feel that your organization treats you with compassion?	Always	41.24%	113	138.29 (<0.001)	2.01
	Often	23.72%	65		
	Sometimes	26.28%	72		
	Rarely	7.30%	20		
	Never	1.146%	4		
Do you think compassion should be considered a key skill in team building?	Strongly agree	53.65%	147	306.40 (<0.001)	>3.0
	Agree	35.40%	97		
	Neutral	10.22%	28		
	Disagree	0.73%	2		
	Strongly Disagree	0.00%	0		
Which of the following aspects do you believe are part of demonstrating compassion in a team? Select all that apply	Active Listening	80.66%	221	85.31 (<0.001)	1.34
	Empathy	65.69%	180		
	Supportive Communication	62.41%	171		
	Understanding Personal Circumstances	50.36%	138		
	Providing Assistance	48.54%	133		
	Offering Encouragement	41.97%	115		
How satisfied are you with the level of compassion shown by your team members?	Very satisfied	42.34%	116	190.48 (<0.001)	3.021
	Satisfied	35.77%	98		
	Neutral	17.52%	48		
	Dissatisfied	3.28%	9		
	Very Dissatisfied	1.09%	3		
Do you believe that showing compassion in team relationships leads to better team performance?	Strongly agree	59.49%	163	363.44 (<0.001)	>3.0
	Agree	32.48%	89		
	Neutral	7.66%	21		
	Disagree	0.36%	1		
	Strongly Disagree	0.00%	0		

### Path analysis

Based on survey no. 2 (*N* = 274), the path analysis revealed a coherent and statistically robust model linking demographic variables, attitudinal beliefs, behavioral recognition, experiential satisfaction, and perceived team outcomes. Gender was excluded due to non-significance, while age emerged as a fundamental predictor: older respondents were more likely to rate compassion as important in team relationships (β = 0.41) and report higher satisfaction with compassion shown by team members (β = 0.36). Perceived importance of compassion acted as a central mediator, predicting endorsement of compassion as a key team-building skill (β = 0.52), which was associated with satisfaction with team compassion (β = 0.48). Satisfaction strongly predicted belief in compassion’s impact on team performance (β = 0.55), reinforcing the experiential-performance linkage. Attitudinal endorsement also shaped behavioral awareness, predicting recognition of compassionate behaviors such as empathy and active listening (β = 0.39). Organizational compassion, defined as being treated compassionately during crises, was predicted by perceived importance (β = 0.44) and contributed to satisfaction (β = 0.50). Religious affiliation significantly influenced workplace attitudes, with moderate to strong effects across domains: team compassion importance (β = 0.52), organizational compassion (β = 0.45), team-building endorsement (β = 0.48), behavioral recognition (β = 0.39), satisfaction (β = 0.46), and performance belief (β = 0.51). Overall, the model delineates a structured pathway from demographic identity through attitudinal beliefs and behavioral recognition to experiential satisfaction and team performance, supported by large effect sizes (Cohen’s w = 1.34–3.0) and highly significant chi-square values (all *p* < 0.001). Model fit was strong (RMSEA ≈ 0.05), confirming the structural validity of compassion as a relational and performance-enhancing construct in team environments.

### Qualitative text analysis in study no. 2: compassion in organizations (open-ended questions)

**Figure 4:**
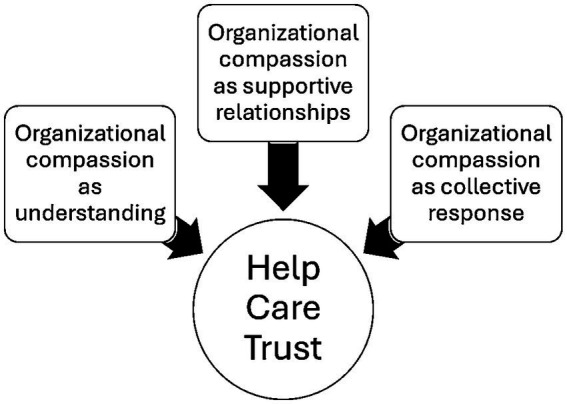
Themes in organizational compassion.

In study no. 2, participants were asked: “Can you provide an example of a time when your organization treated you with compassion during a crisis or complex situation?” The objective of this survey was to examine the role of compassion in team dynamics, crisis response, workplace satisfaction, and organizational performance. Their responses revealed three interconnected themes. The first theme, organizational compassion as understanding, reflected the importance of empathy and recognition of individual circumstances, illustrated by the account “when I had an illness and they were very understanding.” The second theme, organizational compassion as supportive relationship, emphasized tangible assistance and care, captured in the response “helped with accident expenses.” The third theme, organizational compassion as collective response, highlighted coordinated action at the group level, exemplified by the statement “we had a co-worker die during her shift. Management pulled staff from other departments to allow our staff to leave.” Taken together, these themes demonstrate that organizational compassion is expressed through understanding, practical support, and collective action, each contributing to resilience, trust, and well-being in the workplace (Figure 4).

### Set theory model from survey no. 2

We identified three sets of Organizational Compassion (OC): OC₁ = Compassion as Understanding {empathy, patience, emotional recognition}, OC₂ = Compassion as Supportive Relationship {financial assistance, personal support, relational care}, and OC₃ = Compassion as Collective Response {team coordination, crisis adaptation, structural flexibility}. Their intersection, OC₁ ∩ OC₂ ∩ OC₃ = {help, care, trust}, signifies the shared ethical foundation of organizational compassion, while the union OC₁ ∪ OC₂ ∪ OC₃ captures the complete scope of compassionate practice. The synthesis function Compassion = *f*(OC₁, OC₂, OC₃) combines emotional sensitivity, practical support, and coordinated action, resulting in multidimensional organizational behavior. Survey 2 confirmed that this model improves well-being and public health: the intersection fosters emotional safety and cohesion, while the union reflects the range of compassionate responses in crises. Overall, Compassion = *f*(OC₁, OC₂, OC₃) encourages resilience, ethical conduct, and relational stability, reinforcing compassion’s role as a public health asset within organizational settings.

### Result from survey no. 3: perceived compassion in organizations and role of religion or common sense

The research hypothesis was that employees who perceive high levels of compassion within organizational teams, especially during crises, are more likely to report enhanced job satisfaction, well-being, and team performance. The survey (population: *N* = 285) explored perceptions of compassion within workplace relationships, focusing on its connection to religion, common sense, and personal experiences. Gender distribution was balanced (male: 48.08%, female: 51.15%, other/prefer not to answer: 0.76%), with a significant χ^2^ = 232.59 (*p* < 0.001, w = 4.21), suggesting notable variation in gender-related responses. Age distribution showed a concentration in mid-career professionals, with 35–44 years (33.09%) and 45–54 years (31.27%) forming the majority, while younger (18–24: 2.91%) and older (65+: 1.82%) groups were less represented (χ^2^ = 207.06, *p* < 0.001, w = 3.25). Religious affiliation was distributed within Christianity (40.35%), Islam (12.98%), Buddhism (16.14%), Hinduism (10.53%), Judaism (11.58%), and Other (8.24%) (χ^2^ = 97.79%; *p* < 0.001, w = 0.5). Regarding the basis for understanding compassion within organizations, respondents primarily relied on common sense (34.39%), followed closely by religious interpretation (30.18%) and personal experiences (24.56%), while company culture played a minor role (10.18%) (χ^2^ = 114.03, *p* < 0.001, w = 1.63). A majority (58.95%) felt their religion or faith could guide their application of compassion in the workplace, whereas 28.77% disagreed, and 12.28% found the question not applicable (χ^2^ = 95.76, *p* < 0.001, w = 1.42) (Table 4).

**Table 4:** Survey no. 3 – perceived compassion in organizations and role of religion or common sense (multiple-choice questions).

Survey no. 3 (*N* = 285)	Answer choice	Responses (%)	*N*	χ^2^ (GFI) (significance *p*)	Effect size Cohen’s w
Gender	Male	48.08%	134	232.59 (<0.001)	4.21
	Female	51.15%	133		
	Other	0.38%	1		
	Prefer not to answer	0.38%	1		
Age	Under 18	0.73%	2	207.06 (<0.001)	3.25
	18–24	2.91%	8		
	25–34	15.64%	43		
	35–44	33.09%	91		
	45–54	31.27%	86		
	55–64	14.55%	40		
	65+	1.82%	5		
Religious affiliation	Christianity	40.35%	115	97.79 (*p* < 0.001)	0.5
	Islam	12.98%	37		
	Buddhism	16.14%	46		
	Hinduism	10.53%	30		
	Judaism	11.58%	33		
	Other	8.42%	24		
When you consider compassion within your team and organization, what aspects do you primarily focus on?	Religious interpretation	30.18%	86	114.03 (<0.001)	1.63
	Common sense	34.39%	98		
	Personal experiences	24.56%	70		
	Company culture	10.18%	29		
	Other	0.7%	2		
Do you think your religion or faith could offer guidance on interpreting or applying compassion in your workplace?	Yes	58.95%	168	95.76 (<0.001)	1.42
	No	28.77%	82		
	Not applicable	12.28%	35		

### Path analysis

Based on survey no. 3 (*N* = 285), the path analysis revealed a statistically significant model linking demographic identity, interpretive focus, and religious applicability of compassion in workplace settings. Gender was statistically significant (χ^2^ = 232.59, *p* < 0.001, Cohen’s w = 4.21) but excluded as a non-predictive factor. Religious affiliation showed a moderate positive association with prioritizing religious interpretation or personal experience as compassion anchors (β = 0.41). Age emerged as a strong predictor (χ^2^ = 207.06, *p* < 0.001, w = 3.25), with older respondents more likely to emphasize religious interpretation or common sense over personal experience or company culture (β = 0.44). Interpretive preferences mediated belief in religion as workplace guidance (β = 0.52), supported by strong reciprocal effects (Cohen’s w = 1.63 and 1.42). Overall, the model delineates a pathway from age → interpretive focus → religious applicability, highlighting demographic maturity and moral orientation in shaping compassion. Religious affiliation further predicted compassion’s perceived importance in team building, recognition of supportive behaviors, satisfaction with team compassion, and belief in its impact on performance. Compassionate attitudes were closely tied to organizational care during crises, highlighting compassion’s role in effective team dynamics. Model fit was strong (RMSEA ≈ 0.05, values < 0.06 indicating good fit), confirming the structural validity of the pathway.

### Qualitative text analysis in survey no. 3: compassion, religion, and organizations (open-ended questions)

**Figure 5:**
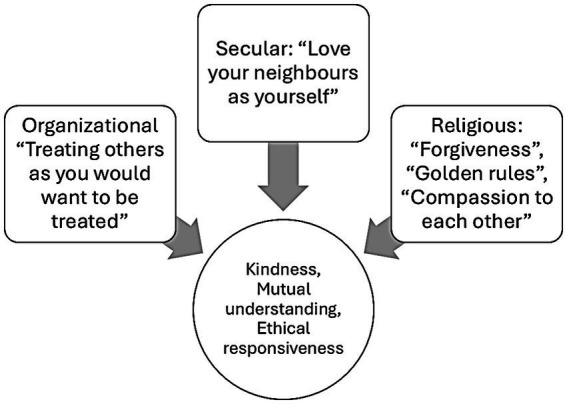
Compassion, organization and religion.

In survey no. 3, participants were asked: “When you consider compassion within your team and organization, what aspect do you primarily focus on?” The objective of this survey was to explore how compassion shapes leadership, team relationships, workplace satisfaction, and collaborative performance across groups. Responses revealed two main themes. The first, reciprocity, highlighted mutual respect and fairness, illustrated by the statement “treating others as you would want to be treated.” The second, understanding, emphasised the importance of empathy and recognizing others’ perspectives, simply expressed as “understanding.” Participants were also asked: “Do you think that your religion or faith could offer guidance on interpreting or applying compassion in your workplace?” Those who answered affirmatively described a variety of influences. The theme of religious guidance included charity, kindness, patience, forgiveness, and love, with references to key teachings such as the Golden Rule and the Ten Commandments. Some responses included “love one another,” “love your neighbor as yourself,” and “religion can help you handle situations better.” Others stressed values like charity, empathy, and active listening, noting that faith traditions encourage approaching challenges with compassion and supporting one another. As one participant explained, “share the same love Jesus Christ showed us and compassion with each other,” while another highlighted that “our work value emphasis on empathy, kindness, and understanding” and fosters a compassionate workplace culture. Overall, these themes show that compassion in organizational settings is understood both as a relational practice of reciprocity and understanding and as a value system enriched by religious or faith-based guidance, which offers moral charity and promotes kindness, patience, and collective support (Figure 5).

### Set theory model from survey no. 3

This model used set theory to formalize compassion in organizational contexts shaped by ethical and religious principles. Three domains were defined: organizational (𝑂 = reciprocity, empathy, active listening), religious (𝑅 = charity, forgiveness, Golden Rule), and secular ethics (𝐸 = universal moral principles such as fairness and treating others well). Their intersection, 𝐶 = 𝑂 ∩ 𝑅 ∩ 𝐸, represented the shared core of compassion—kindness, mutual understanding, and ethical responsiveness—essential for leadership and team cohesion. The union, 𝑂 ∪ 𝑅 ∪ 𝐸, captured the full landscape of compassion, including organizational practices, religious directives, and ethical imperatives. Religious teachings influenced organizational behavior (𝑅 → 𝑂), while ethical principles guided team functioning (𝐸 → 𝑂). Jointly, R ∪ E → O illustrated how integrated moral and spiritual foundations shape compassionate leadership. The synthesis function, Compassion = *f*(𝑂, 𝑅, 𝐸), expressed how these domains interact to produce environments of charity, empathy, and ethical responsiveness. Survey 3 confirmed that received compassion—through O, R, and E—emerges from their intersection as a unified framework that enhances psychological safety, moral charity, and relational well-being, affirming compassion’s role in public health and organizational resilience.

### Aggregated results

Across all three surveys, the integrated path and set-theory analyses uncovered a clear, statistically significant progression linking demographic factors to compassion-related beliefs, behaviors, and workplace experiences. Age consistently emerged as the most influential predictor, with older respondents more likely to value compassion, recognize its underrepresentation in media, and prioritize religious or commonsense interpretations. Religious affiliation provided a distinct moral and interpretive perspective, influencing how compassion was understood, endorsed in education, and perceived in media, team dynamics, and crisis response. Notably, receiving compassion from others was not viewed uniformly: within religious frameworks, compassion was often understood as divine grace, moral duty, or communal solidarity, whereas within secular frameworks, it was framed as ethical care, humanistic responsibility, or fairness. These dual interpretive pathways highlight compassion’s versatility as both a spiritual and civic resource. Attitudinal beliefs—including the importance of compassion, its teachability, and moral foundation—served as mediators, linking demographic maturity and moral orientation with lived organizational and societal experiences. Respondents who regarded compassion as teachable and morally grounded were more likely to recognise compassionate behaviors, report satisfaction with team interactions, and affirm its influence on performance and social life. Gender showed some statistical variation but played a minor role overall and was therefore excluded from the final models. At organizational level, compassion translated into tangible outcomes. Recognition of compassionate behaviors, satisfaction with team dynamics, and confidence in compassion’s impact on performance were consistently reported. Receiving compassion within workplace settings was associated with reduced stress, increased resilience, and higher satisfaction, reinforcing its restorative function in professional environments.

Beyond the workplace, compassion emerged as a relational ethic defined by empathy, non-judgment, altruism, supportive relationships, and collective response. Experiencing compassion from others validated identity, strengthened psychological well-being, and reinforced trust and cohesion within communities. Religious frameworks often reinforced compassion as a sacred obligation, while secular frameworks emphasized its role in sustaining fairness, cooperation, and civic responsibility. Together, these perspectives enriched compassion’s meaning and broadened its impact. Finally, the integrated models demonstrated compassion’s reach across multiple domains of influence. At the personal level, receiving compassion enhanced emotional regulation, resilience, and satisfaction, with religious interpretations deepening its spiritual resonance and secular interpretations reinforcing its ethical charity. Institutionally, compassion embedded in organizational settings, education, and organizational practice improved outcomes and equity, while also legitimizing compassion as a professional standard. At the societal level, compassion strengthened public health, media representation, and community cohesion, positioning it as a shared moral and operational construct that sustains healthier individuals, stronger institutions, and more cohesive communities (Table 5).

**Table 5:** Summative results.

Section	Variables	Impact on personal and societal well-being
Demographic predictors	Age drives compassion values; religion shapes interpretation; gender is excluded.	Age adds moral charity; religion frames received compassion as grace, duty, or solidarity, strengthening resilience and identity.
Attitudinal beliefs (mediators)	Beliefs in compassion’s importance, teachability, and moral grounding bridge demographics with outcomes.	Seeing compassion as teachable enhances emotional regulation; societal embedding normalizes compassion in education and discourse.
Organizational outcomes	Compassion attitudes translate into recognition, team satisfaction, and performance impact.	Receiving compassion at workplace reduces stress and builds resilience; compassionate workplaces foster healthier institutions.
Relational ethic	Compassion is defined by empathy, non-judgment, altruism, supportive ties, and collective response.	In the individual, compassion from others validates identity and well-being; in social relationships it builds trust, cohesion, and resilience in communities.
Domains of influence	Compassion spans personal, institutional, and societal domains.	Receiving compassion boosts emotional balance and satisfaction; institutions and societies gain equity, cohesion, and public health benefits.

## Discussion and conclusion

All objectives and hypotheses were confirmed, illustrating compassion’s indirect yet significant role in enhancing personal well-being and public health. Participants consistently interpreted compassion through religious, secular, and intuitive frameworks, highlighting its pluralistic relevance. Compassionate acts were recognized across family, workplace, healthcare, and community settings, functioning as relational mechanisms that foster resilience and social support. These interpretations indirectly support the current trend in considering receiving compassion as a factor contributing to personal well-being and broader public health outcomes, by reinforcing resilience, fostering social cohesion, and shaping organizational cultures that embed compassionate practices. At the broader level, compassion indirectly shaped organizational culture and community resilience, supporting the institutionalization of compassionate practices. Collectively, findings demonstrate compassion’s power to advance public health outcomes through its relational and interpretive impact. Therefore, the results confirm previous research and extend understanding of compassion’s global impact, demonstrating its influence both at the micro level in interpersonal relationships and at the macro level as a sustaining force within organizations and societies (68–78).

Scholarship on compassion underlines its dual function as both an interpersonal virtue and a systemic resource. Research on bidirectional compassion indicates that giving and receiving support are mutually reinforcing, fostering trust, resilience, and psychological safety (79). Receiving compassion specifically alleviates stress, reduces isolation, and aids recovery, thereby enhancing individual well-being and collective health (79). Crawford and colleagues expand this perspective by analyzing compassionate language in acute mental health care, demonstrating how discourse influences therapeutic relationships and creates environments where patients feel heard, valued, and supported (79). Collectively, these insights emphasize compassion’s vital role in fostering resilience and health across personal and institutional settings (79). Brown and colleagues highlight practical compassion, where everyday gestures operationalise care and affirm dignity (80). These micro-level acts, when received, strengthen emotional resilience and improve adherence to treatment, with downstream effects on public health indicators such as reduced hospital readmissions and improved community trust in healthcare systems. The notion of compassionate environments further emphasises organizational culture, team cohesion, and institutional support as critical to sustaining compassion (81, 82). When healthcare professionals themselves experience compassion, burnout is reduced, staff retention improves, and the quality of care increases (78). Compassionate design incorporates compassion into social environments, systems, and procedures, normalizing it as a collective resource that boosts resilience and promotes equitable, sustainable health outcomes (78).

## Limitations

This study offers valuable insights into how receiving compassion influences mental well-being and organizational climate, but several limitations should be recognized. The sample was limited to working-age adults in the United States, limiting its applicability across cultures, generations, and institutional settings. Although the mixed-method design enhanced interpretation, reliance on self-reported data introduces biases such as social desirability and retrospective distortion. The absence of longitudinal data hindered the evaluation of long-term effects on well-being and collaboration, while the exploration of religious and secular ethical frameworks did not fully disentangle their individual impacts or consider intersectional factors like race, gender, religion, and socioeconomic status. Furthermore, the lack of observational or behavioral data limits the validation of reported experiences. Future research should adopt cross-cultural and longitudinal designs, incorporate behavioral methods to triangulate self-reports, and investigate how intersectional identities and ethical frameworks influence reception.

Several types of generalizability strengthen the contribution of this study. Statistical generalizability is supported by findings from a large U.S. working-age sample, providing a foundation for future cross-national or multi-site research. Ecological generalizability highlights compassion’s adaptability across workplaces, schools, and community organizations, underscoring its role as relational infrastructure. Cross-cultural transferability points to the potential for replication in diverse contexts to test whether predictors such as age and religious affiliation are universal or vary by culture. Intersectional transferability emphasizes relevance across overlapping identities, including race, gender, socioeconomic status, and religion. Temporal generalizability establishes a basis for examining whether compassion’s impact on well-being and organizational performance persists across life stages and changing social conditions. Finally, conceptual or theoretical transferability ensures that the relational framework of compassion can inform public health, ethics, and community resilience, positioning the study as a versatile and impactful contribution.

## Data Availability

The raw data supporting the conclusions of this article will be made available by the authors, without undue reservation.
