# Supplementary material for: Mapping compassion in the general adult population: religious and secular compassionate acts in social relationships and organizational culture
Source: Front Public Health. 2026 Jan 21;13:1704798. doi: 10.3389/fpubh.2025.1704798 (PMC12867770; doi:10.3389/fpubh.2025.1704798)
Supplement: Supplementary file 1 [file Data_Sheet_1.docx]

# STROBE Checklist for Observational Studies

| Item | Recommendation | Covered |
| --- | --- | --- |
| Title and Abstract |  | X |
| 1(a) | Indicate the study’s design with a commonly used term in the title or abstract | X |
| 1(b) | Provide an informative and balanced abstract summarising methods and findings | X |
| Introduction |  |  |
| 2 | Explain the scientific background and rationale | X |
| 3 | State specific objectives and any prespecified hypotheses | X |
| Methods |  |  |
| 4 | Present key elements of study design early | X |
| 5 | Describe setting, locations, and relevant dates | X |
| 6(a) | Provide eligibility criteria and selection methods | X |
| 6(b) | Provide matching criteria where applicable | X |
| 7 | Define outcomes, exposures, predictors, confounders, and effect modifiers | X |
| 8* | Provide data sources and assessment methods | X |
| 9 | Describe efforts to address potential bias | X |
| 10 | Explain how study size was determined | X |
| 11 | Describe handling of quantitative variables | X |
| 12(a) | Describe all statistical methods including confounding control | X |
| 12(b) | Describe subgroup and interaction analyses | X |
| 12(c) | Explain handling of missing data | X |
| 12(d) | Describe follow-up, matching, or sampling strategy as applicable | X |
| 12(e) | Describe sensitivity analyses | X |
| Results |  |  |
| 13(a)* | Report numbers at each study stage | X |
| 13(b) | Provide reasons for non-participation | X |
| 13(c) | Consider flow diagram | X |
| 14(a)* | Provide participant characteristics and confounder information | X |
| 14(b) | Indicate missing data per variable | X |
| 14(c) | Summarise follow-up time for cohort studies | X |
| 15* | Report outcome data | X |
| 16(a) | Provide unadjusted and adjusted estimates with precision | X |
| 16(b) | Report category boundaries for categorised variables | X |
| 16(c) | Translate relative risk into absolute risk where relevant | X |
| 17 | Report additional analyses including subgroup and sensitivity analyses | X |
| Discussion |  |  |
| 18 | Summarise key results with reference to objectives | X |
| 19 | Discuss limitations including potential bias and imprecision | X |
| 20 | Provide cautious interpretation considering objectives, limitations, and evidence | X |
| 21 | Discuss generalisability of findings | X |
| Other Information |  |  |
| 22 | Provide funding sources and role of funders | X |
